# Circadian Disruption in Critical Illness

**DOI:** 10.3389/fneur.2020.00820

**Published:** 2020-08-11

**Authors:** Aesha M. Jobanputra, Matthew T. Scharf, Ioannis P. Androulakis, Jag Sunderram

**Affiliations:** ^1^Division of Pulmonary, Critical Care, and Sleep Medicine, Department of Medicine, Rutgers Robert Wood Johnson Medical School, New Brunswick, NJ, United States; ^2^Department of Neurology, Rutgers Robert Wood Johnson Medical School, New Brunswick, NJ, United States; ^3^Biomedical Engineering Department, Rutgers University, Piscataway, NJ, United States; ^4^Chemical and Biochemical Engineering Department, Rutgers University, Piscataway, NJ, United States; ^5^Department of Surgery, Rutgers Robert Wood Johnson Medical School, New Brunswick, NJ, United States

**Keywords:** circadian rhythm, critical illness, sleep, ICU, trauma

## Abstract

Circadian rhythms play a vital role in metabolic, hormonal, and immunologic function and are often disrupted in patients in the ICU. Circadian rhythms modulate the molecular machinery that responds to injury and illness which can impact recovery. Potential factors contributing to the alteration in circadian rhythmicity in intensive care unit (ICU) patients include abnormal lighting, noise, altered feeding schedules, extensive patient care interactions and medications. These alterations in circadian rhythms in ICU patients may affect outcomes and therefore, normalization of circadian rhythmicity in critically ill patients may be an important part of ICU care.

## Introduction: Circadian Rhythms

Organisms evolved to anticipate and adapt to the cyclic nature of the environment on earth, most notably diurnal variations in sunlight and temperature over the earth's 24-h rotation about its axis. An endogenous timing system, known as the circadian rhythm, aligns biological processes such as activity, food intake, alertness, and energy expenditure to this day/night pattern. Environmental cues such as the light/dark cycle, changes in temperature, and availability of food, act as *zeitgebers* (timekeepers*)*, which synchronize the endogenous circadian clock with the external environment. Therefore, the circadian rhythm is the result of an endogenous timing system which is influenced by external stimuli.

The circadian timing system is organized as a hierarchical and interconnected network of clocks which are present in nearly every cell in the body (1). The autonomous oscillation of each of these circadian clocks is driven by transcriptional and translational feedback loops such that each clock can maintain an ~24 h rhythm (2). These endogenous oscillations are maintained unless acted upon by an external influence.

While these clocks are distributed across all cells, in higher organisms including mammals they need to be synchronized within and across tissues and organs. This role is played by the central clock in the suprachiasmatic nucleus (SCN) of the hypothalamus. Since light is the primary *zeitgeber*, the SCN needs to be coupled to the external light/dark cycle. Indeed, the central clock in the SCN receives photic input from retinal ganglion cells in the eye (3, 4) which allows for entrainment of the circadian rhythm to the exogenous light/dark cycle. The SCN, in turn, transduces this information to the peripheral clocks within the central nervous system as well as to other organ systems. Effectively, the SCN does this by controlling timing of feeding and activity cycles as well as regulating the rhythmic release of endocrine hormones (5, 6).

**Figure 1:**
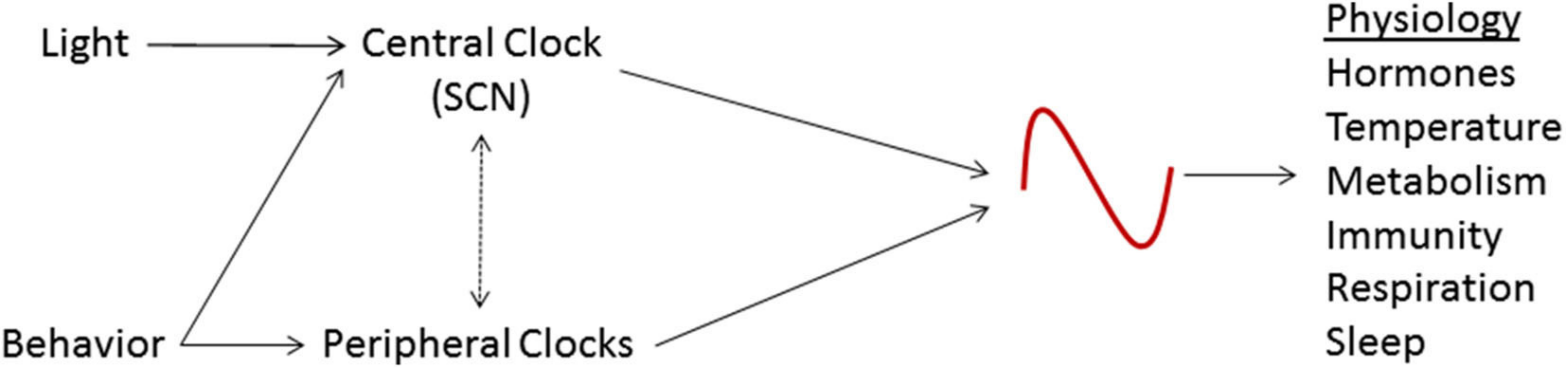
Schematic representation of the circadian system. The suprachiasmatic nucleus (SCN), the central circadian clock, is entrained by light and other stimuli. The SCN coordinates the activity of peripheral clocks and is, in turn, affected by them. These circadian clocks regulate various physiologic processes.

The circadian system causes rhythmic patterns in behavior and physiology in organisms ranging from the simplest to the most complex (7). A variety of physiological activities, including immune response, metabolism, respiration, sleep cycles and endocrine signaling, are modulated by the circadian rhythm (8, 9). It is now well recognized that local and central rhythms must be synchronized to each other as well as to the outside environment to achieve optimal functioning (10). Conversely, situations that lead to the breakdown of circadian coordination or circadian desynchronization can cause significant deleterious consequences (11) (Figure 1).

## Circadian Regulation of the Response to Critical Illness and Trauma

Critical illness is often accompanied by an inflammatory response and this inflammatory response may mediate the recovery from illness (12). Interestingly, markers of the systemic inflammatory response including temperature, respiratory rate, blood pressure, hormones (cortisol, melatonin, leptin, prostaglandins), cytokines, white blood cell and platelet counts, all display a circadian rhythmicity (13). The circadian regulation of the various components of the inflammatory response ensures appropriate timing and maintenance of the immune response and therefore, a dysregulated circadian system, or a stressor to the circadian system at an inopportune time, could alter the immune response and the recovery from injury.

Extensive e*x vivo* and *in vivo* studies have demonstrated the circadian control of the inflammatory response. Keller et al. were among the first to convincingly demonstrate that macrophages in diverse mouse tissues contain autonomous circadian clocks conferring to tissues the ability to sense danger signals, lipopolysaccharides (LPS), in a time-of-day dependent manner (14). They demonstrated that these tissue-specific rhythms are endogenous and not generated by systemic (glucocorticoid) signaling. The implications of this study are significant since the time-of-day-dependent regulation of macrophages could not only explain the diurnal variation in survival rate in endotoxin-induced mouse shock models, but also points to the integration of environmental cues (light, food, activity, etc.) into the immune system response.

Additional evidence for circadian regulation of the immune response was demonstrated by Haimovich et al. who exposed healthy human subjects to endotoxin at either 9 a.m. or 9 p.m. and demonstrated a marked difference in the secretion of inflammatory cytokines and suppression of circadian clock gene expression in peripheral blood leukocytes that was dependent on the time of exposure (15). Further evidence of the host's internal clock influencing the response to a pathogen was shown by Edgar et al. who demonstrated that exposing a host (mouse) to a virus (influenza A and herpesvirus) during the active period results in an attenuated viral replication and infection compared to exposing the host to the virus during the rest phase. They further demonstrated that this time-of-day dependence was abolished in mice with a deletion of a circadian clock gene suggesting that the circadian clock was mediating this response (16). These results demonstrate that the response to a pathogen is influenced by the host's circadian clock. Below are examples of the circadian influence on recovery in patients:

### Burn Healing

Analysis of a burn injury database demonstrated that patients had a faster time to recovery if they were burned during the day rather than at night. To explore this phenomenon, Hoyle et al. demonstrated in mice that wounds incurred during the “active” phase healed faster and more efficiently. They further demonstrated that the cell-autonomous fibroblast circadian clocks drive the rhythmic regulation of actin-dependent processes including fibroblast invasion which, in turn, drive a “time-of-day” dependence of burn-induced skin wound healing in mice (17). The authors speculated that the circadian regulation of the actin cytoskeleton plays a major role in driving this diurnal dependence.

### Aortic Valve Replacement Surgery

A time-of-day dependence on the recovery from aortic valve replacement surgery was demonstrated in a recent study by Montaigne et al. In a cohort of nearly 600 patients, the incidence of major adverse cardiac events following surgery was lower in patients operated upon in the afternoon compared to the morning during a 500 day follow-up period. The authors subsequently randomized 88 patients to receive aortic valve replacement surgery in either the morning or afternoon and found that perioperative myocardial injury, as measured by Troponin T, was lower in patients who were operated upon in the afternoon compared to the morning. They further demonstrated a morning-afternoon variation in hypoxia-reoxygenation tolerance concominant with changes in circadian gene expression in human myocardium with expression of *Rev-Erb*α, a key circadian gene, being highest in the morning. In a mouse model of hypoxia-reoxgenation myocardial injury, *Rev-Erb*α gene deletion or antagonist treatment reduced myocardial injury (18). These results suggest that the high expression of *Rev-Erb*α in the morning increases the risk of myocardial injury. More broadly, these results are an example of clinically significant “morning vs. afternoon” variation in surgical outcomes effectively orchestrated by underlying circadian regulatory mechanisms.

### Recovery From Blunt Trauma

To examine the time-of-injury dependence on the recovery from blunt trauma, Zaaqoq et al. studied two propensity-matched cohorts of survivors of blunt trauma, those who were injured either in the day or at night. Patients who were injured at night had a longer length of stay both in the hospital and the intensive care unit and required greater surgical interventions and trauma management. The authors measured a number of inflammatory mediators and showed marked differences in the evolution of the inflammatory trajectories and immune responses that were dependent on whether the patient was injured during the day or night (19). These results suggest that the recovery from an injury is dependent on the circadian regulation of inflammatory mediators such that injuries that occur when the circadian system is better aligned heal better.

## Circadian Rhythms in the Disruptive ICU Environment

**Figure 2:**
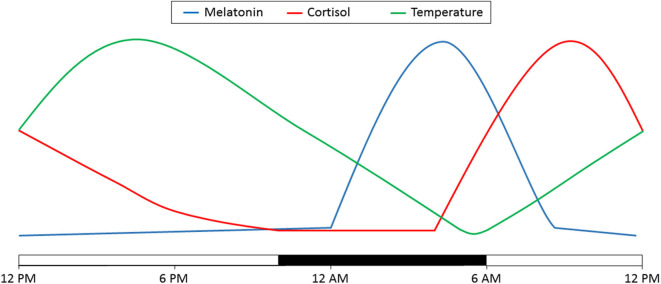
Diurnal variations in melatonin, cortisol and temperature. Depicted here is the variation over 24 h. Time of day is denoted on the bottom. The light bars represent time subjects are normally awake and the dark bar represents a typical 8 h sleep time. Adapted from (20).

Considerable evidence is emerging that patients in the ICU exhibit profound circadian rhythm disruptions. Since direct measurement of the circadian clock in humans is not generally feasible, surrogates of the circadian clock are often used to assess circadian rhythmicity. Three commonly assessed surrogates are core body temperature (CBT), melatonin or cortisol. Under normal circumstances, CBT exhibits a peak during the active period and a nadir at the end of the rest period. Conversely, melatonin exhibits a peak during the rest period while cortisol demonstrates a peak during the inactivity to activity transition period (Figure 2) (20).

**Figure 3:**
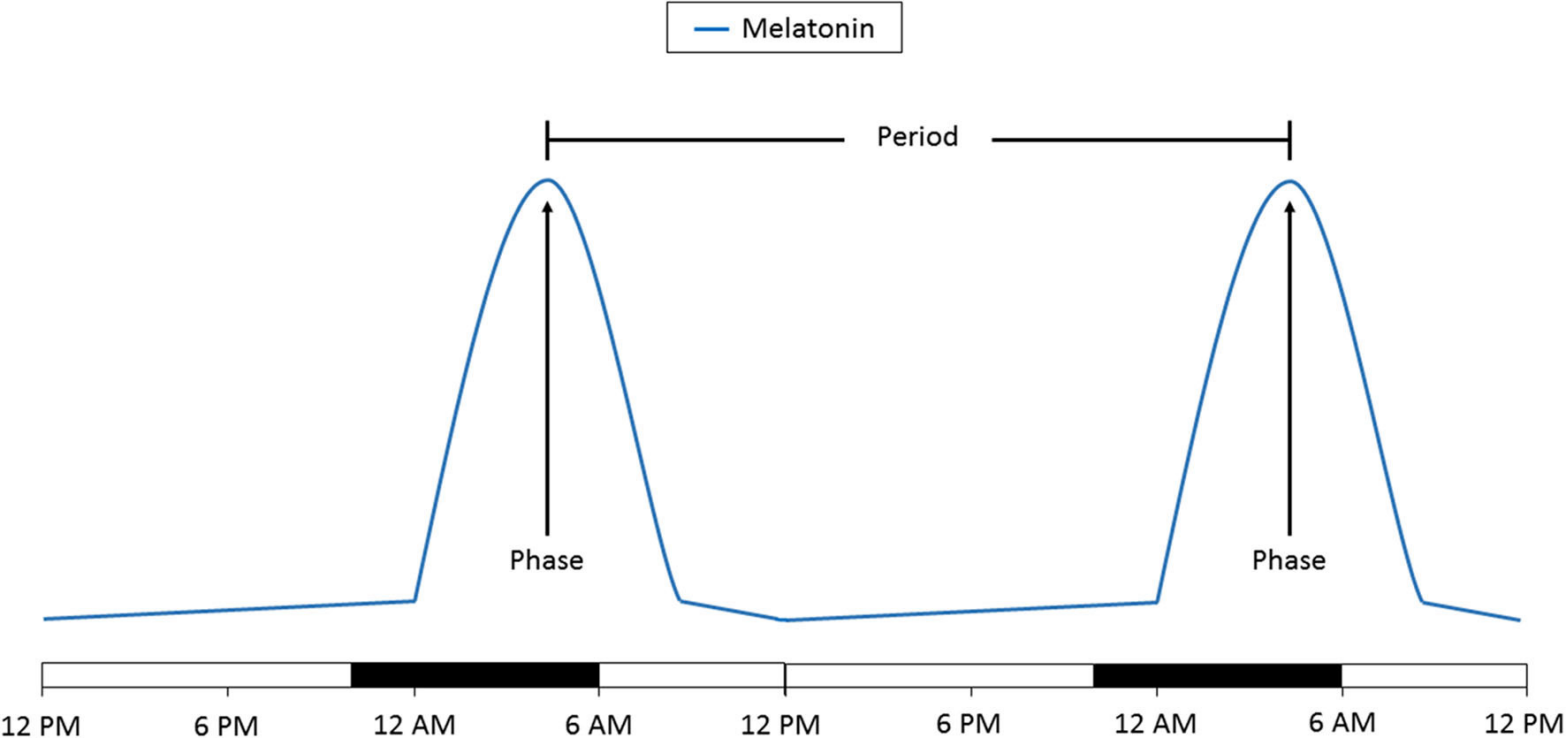
Schematic representation of the change in melatonin levels over 48 h. The time of day at which peak melatonin levels are reached is the phase. The amount of time between two phases is the period. A change in the phase indicates a time shift in the circadian clock. A change in the period to more or <24 h indicates that the circadian clock is no longer being entrained. Time of day is denoted on the bottom. The light bars represent time subjects are normally awake and the dark bars represents a typical 8 h sleep time.

Any circadian signal is broadly characterized by two features: its period and its phase. Broadly, the period can be defined as the time interval between successive occurrences of the same measurement and the phase can be described as the relative location of a minimum or maximum value during the period. Measurement of the phase, which can be the peak or nadir, can be used as a measure of the timing of the circadian clock (Figure 3). When looking at a population, the distribution of phases can be used to determine how consistently that population is timed to the external environment. A wide distribution of phases suggests that the individuals within that population are timed differently to the environment and may even be completely uncoupled to external stimuli.

A number of studies have assessed the timing of the circadian clock in patients in the ICU. Gazendam et al. assessed the nadir of the CBT in patients in the ICU. They found that patients in the ICU did exhibit a nadir in CBT, but instead of it occurring during the early morning hours, the nadirs in different patients were distributed throughout the entire 24 h day (21). In fact, it was further shown that the patient's APACHE III score, an indicator of the severity of critical illness, correlated well with the extent of this shift. This study suggests that circadian rhythms are present in ICU patients and that the central circadian clock is functional, but the clocks are not properly entrained to the external environment.

Interestingly, Gehlbach et al. arrived at a similar conclusion using a different circadian biomarker, urinary secretion of aMT6, a product of the metabolism of melatonin. They studied a cohort of mechanically ventilated patients receiving continuous intravenous sedation. They found that these ICU patients generally had preserved diurnal variation of aMT6, however, most of the patients had misaligned timing of aMT6 secretion with a wide distribution of phases (22). Similarly, cortisol levels measured in trauma patients in an ICU displayed a circadian rhythmicity, but the phase distribution was very wide. This was more marked in patients with sepsis compared to those without sepsis (23).

These observations dovetail with the time-of-day dependent mortality pattern observed in the ICU. Sudden cardiac death in the general population shows a peak in the early morning hours (24). In one study, the morning peak in mortality was observed in non-ICU patients, but was not observed in patients in the ICU. Furthermore, there was no clear mortality peak observed at any time-of-day in the ICU patients (25). If ICU patients display a broad distribution of phases and therefore are not well-aligned to the external environment, one would expect that there would be no observable time-of-day peak in mortality.

These studies suggest that ICU patients maintain circadian rhythms, however, these rhythms become uncoupled to the external environment. This could be due to disruptions in the signals which normally entrain the circadian clock (see below), but it could also have to do with other temporal factors such as timing of onset of a critical event (e.g., septic shock or injury time) which may uncouple the clock to the external environment (17, 19, 26).

## Causes of Circadian Disruption in the ICU

Patients in the ICU are subject to extensive monitoring and interventions throughout the day and night. While critical illness itself may cause circadian rhythm disruption, it is likely that the round-the-clock care provided in the ICU also disrupts the circadian rhythm. Possible contributing factors are discussed below:

### Light

Under normal circumstances, people are exposed to much more light during the day and during waking hours. Danielson et al. monitored light intensity in individual patient rooms in an ICU and found a wide range of light patterns from constant darkness to light/dark patterns that were completely inverted. They also noted that the average light intensity was low. Interestingly, patients and families did not rate the light levels as being too low suggesting that this problem may be underappreciated patients and their families (27) but see (28). Light is the primary *zeitgeber* and the absence of normal light intensity and light exposure at inappropriate times could be a major cause of the abnormal circadian rhythms that are observed in patients in the ICU.

### Noise

Sources of noise in the ICU include conversations, various patient care equipment alarms, moving of equipment and the mobilization of clinical staff to attend to patient care urgencies and emergencies. The Environmental Protection Agency recommended in 1974 that noise levels in hospitals should be <45 decibels (dB) in the day and <35 dB at night. However, one study found median noise levels in the ICU around 50 dB with little difference between days and nights (27). Furthermore, noise levels at night at times exceeded 80 dB (27, 28). Loud noise occurring at night as well as noise occurring at similar levels between the day and night could disturb both sleep and circadian rhythms.

### Medication

Medications used for sedation such as benzodiazepines or opiates can alter sleep-wake patterns which could affect circadian rhythmicity. Another class of medications, corticosteroids, are commonly used in the ICU for a number of conditions and may significantly affect the circadian clock. Exogenous administration of corticosteroids, including synthetic analogs of cortisol, can interfere with the endogenous rhythm of cortisol release. Depending on the time of day, corticosteroid infusion can lead to either suppression or induction of endogenous cortisol production (29). Since cortisol levels fluctuate during the day in a circadian-dependent manner (see above) and cortisol plays an essential role in the link between the central and peripheral clocks and in coordinating the host's circadian rhythms (30), disruption of this system could lead to profound physiologic consequences. This could manifest as a dysregulated circadian clock, but more significantly, it can disrupt an important mechanism by which the circadian clock regulates diffuse processes throughout the body.

### Feeding

Under normal conditions, eating times are regulated by the master circadian clock, the SCN. When food availability is disconnected from the master clock, the metabolic processes that are regulated by nutritional inputs are also dysregulated. This misalignment has been associated with a number of metabolic disorders (31). In fact, feeding patterns need to exhibit proper synchrony with SCN-driven endocrine signals (32) since food intake establishes rhythms in metabolically active peripheral tissues and organs which need to be maintained in proper alignment with the central clock (33). Despite the fact that the scientific literature overwhelmingly supports the idea of properly aligning meals times with internal rhythms (34), patients in the ICU may not be fed in this manner. Many patients in the ICU are maintained on enteral nutrition and that nutrition may be given in bolus feeds at various time intervals or may even be administered continuously during the day and night. Feeding out of line with the circadian rhythm may contribute to further circadian disturbance and contribute to other physiologic problems.

### Patient Care Interactions

Due to the high level of care administered in an ICU, there are frequent patient interactions related to medication administration, checking blood pressures, phlebotomy, wound care and patient hygiene among other reasons. Studies by Tamburri et al. and Celik et al. have reported an average of 40–50 patient care interactions per patient per night (35, 36). Meyer et al. reported that patient care interactions in the ICU occurred at least hourly (28). These studies demonstrate the high frequency of patient care interactions in the ICU at night during what is considered a normal sleep time. The frequency of these interactions can lead to sleep disturbance and may also cause disruption of the circadian clock.

## Circadian Rhythm Restoration to Improve Outcome

Given the circadian rhythm disturbance that is present in patients in the ICU and the deleterious consequences of circadian rhythm disruption, a number of studies have assessed whether restoration of circadian rhythmicity can improve outcomes. Potential therapies include both modification of the external environment as well as medication administration.

Patients in the ICU often have a circadian phase shift and exposure to light, often the major *zeitgeber*, in an unnatural manner (see above). Gehlbach et al. assessed ICU patients with a phase delay and treated these patients with light box exposure from 9 a.m. to noon. They measured circadian rhythmicity with the melatonin metabolite, urinary excretion of aMT6. With just 2 days of light treatment, there was a phase advance and an increased amplitude of the aMT6 peak suggesting that the circadian rhythm of these patients was being entrained to a more “normal” rhythm (37). Despite being a small, pilot study, the results demonstrate the feasibility of a simple intervention to restore proper rhythmic characteristics of the central clock in ICU patients. Interestingly, morning light exposure has been shown to positively impact delirium in three out of five studies (38–42) suggesting that this treatment may improve not just circadian rhythmicity, but also a medical problem that is common in ICU patients.

Timed administration of melatonin can be used to treat circadian rhythm disorders (43, 44). The time of day that the melatonin is administered has to be timed to the desired circadian rhythm. Administration of melatonin in the evening, which may facilitate a “normal” melatonin peak in the early morning hours, was shown to improve markers of sleep in two out of three studies and reduced delirium in three out of four studies (45–51). These observations have led to the suggestion that light therapies and melatonin administration should be tested in full-scale clinical trials in ICU patients (52).

Tailoring administration of medications which are commonly used in the ICU to preserve or enhance normal circadian rhythmicity is another possible approach to improve outcomes. As discussed earlier, use of corticosteroids may alter the endogenous rhythm of cortisol release which could affect both the circadian clock itself and the circadian coordination of various physiologic processes. Two studies assessed whether intermittent vs. continuous administration of hydrocortisone, a corticosteroid, improved reversal of shock in ICU patients. A retrospective study did not show a difference in shock reversal with intermittent vs. continuous administration of hydrocortisone (53), but a small randomized open-label trial showed that intermittent administration of hydrocortisone was associated with improved shock reversal compared to continuous infusion (54). Although it is likely that these protocols affected circadian rhythmicity, this was not measured in either of these studies. Clearly, these issues require further elucidation and it is possible that use of corticosteroids in a manner that potentiates natural circadian rhythmicity may improve outcomes (55).

Enteral nutrition administration in a manner which mimics normal eating could potentially improve outcomes by aligning feeding to the circadian rhythm. Enteral nutrition can be delivered continuously or intermittently with various schedules and methods of delivery. Practical factors can play a role in deciding which is used (56). A number of clinical studies have considered the impact of temporal delivery of enteral feeding in critically ill patients (57–64) motivated mostly by the need to address some of the key practical limitations associated with delivering nutritional support rather than exploring nutritional routes as a means to metabolically engage the circadian system. The challenges and opportunities of complex feeding schedules are highlighted in Sunderram et al. (65).

Finally, simple approaches to regulate the external environment are likely to be important. Optimizing the natural light in patient rooms in the ICU, limiting noise during the night, safely limiting patient care interactions during normal sleep times and generally creating an environment that is more conducive to sleep at night should be strongly considered. Further studies are needed to evaluate outcomes after implementation of some of these interventions.

## Conclusion

The circadian system plays a vital role in regulating various physiologic processes. The way the body responds to injury is often dependent on the interaction of the injury with the circadian machinery. Circadian rhythms are often disrupted in patients in the ICU and there are a number of factors that likely contribute to this breakdown. Practices leading to circadian rhythm optimization may improve patient outcomes and implementation of these practices should be incorporated into ICU care.

## Author Contributions

AJ, MS, and IA wrote parts of the manuscript. MS and IA created the figures. AJ, MS, and JS edited the manuscript. All authors contributed to the article and approved the submitted version.

## Conflict of Interest

The authors declare that the research was conducted in the absence of any commercial or financial relationships that could be construed as a potential conflict of interest.
